# Predictors of dementia among the oldest old: longitudinal findings from the representative “survey on quality of life and subjective well-being of the very old in North Rhine-Westphalia (NRW80+)”

**DOI:** 10.1186/s12877-024-05255-z

**Published:** 2024-08-13

**Authors:** André Hajek, Benedikt Kretzler, Steffi G. Riedel-Heller, Razak M. Gyasi, Hans-Helmut König

**Affiliations:** 1 Department of Health Economics and Health Services Research, University Medical Center Hamburg-Eppendorf, Hamburg Center for Health Economics, Hamburg, Germany; 2 Institute of Social Medicine, Occupational Health and Public Health, University of Leipzig, Leipzig, Germany; 3 African Population and Health Research Center, Nairobi, Kenya; 4 National Centre for Naturopathic Medicine, Faculty of Health, Southern Cross University, Lismore, NSW Australia

**Keywords:** Oldest old, Aged, 80 and over, Dementia, Cognitive decline, Depression, Health literacy, Loneliness, Functional impairment, Education, Network size

## Abstract

**Background/Aims:**

Our current study aimed to investigate the determinants of dementia among the oldest old using longitudinal data from a representative sample covering both community-dwelling and institutionalized individuals.

**Methods/Design:**

Longitudinal representative data were taken from the “Survey on quality of life and subjective well-being of the very old in North Rhine-Westphalia (NRW80+)” that surveyed community-dwelling and institutionalized individuals aged 80 years and above (*n* = 1,296 observations in the analytic sample), living in North Rhine-Westphalia (most populous state of Germany). The established DemTect was used to measure cognitive impairment (i.e., probable dementia). A logistic random effects model was used to examine the determinants of probable dementia.

**Results:**

The mean age was 86.3 years (SD: 4.2 years). Multiple logistic regressions revealed that a higher likelihood of probable dementia was positively associated with lower education (e.g., low education compared to medium education: OR: 3.31 [95% CI: 1.10–9.98]), a smaller network size (OR: 0.87 [95% CI: 0.79–0.96]), lower health literacy (OR: 0.29 [95% CI: 0.14–0.60]), and higher functional impairment (OR: 13.45 [3.86–46.92]), whereas it was not significantly associated with sex, age, marital status, loneliness, and depressive symptoms in the total sample. Regressions stratified by sex were also reported.

**Discussion:**

Our study identified factors associated with dementia among the oldest old. This study extends current knowledge by using data from the oldest old; and by presenting findings based on longitudinal, representative data (also including individuals residing in institutionalized settings).

**Conclusions:**

Efforts to increase, among other things, formal education, network size, and health literacy may be fruitful in postponing dementia, particularly among older women. Developing health literacy programs, for example, may be beneficial to reduce the burden associated with dementia.

## Introduction

In high-income countries such as Germany, the population continues to age. The number of the oldest people in particular (80 years and older) is steadily increasing. This stage of life (i.e., 80 years and over) is associated with various challenges. For example, marked decreases in health [1] (such as multimorbidity [2]) often occur, which can hinder social activities [3]. Moreover, other critical life events frequently take place such as the loss of friends and relatives (e.g., spousal loss [4]), nursing home admission [5] or serious falls [6]. Such factors can lead to loneliness [7]. Due to the large number of serious critical life events frequently taking place in this life stage, it is important to take a closer look at individuals aged 80 years and over.

Being 80 years and older is also associated with dementia [8]. Dementia is a progressive cognitive impairment syndrome caused most commonly by Alzheimer’s disease. For example, according to a recent systematic review/meta-analysis, the prevalence of all-type dementia is about 2.7% among individuals aged 65 to 74 years. In contrast, the prevalence of all-type dementia is 15.1% among individuals aged 80 to 89 years and 35.7% among individuals aged 90 to 99 years, and 65.9% among individuals aged 100 years and over [8].

Individuals suffering from dementia are at high risk of feeling lonely [9], having major depression [10] or reporting lower health-related quality of life [11]. Managing daily activities (e.g., complex activities such as handling finances and even more basic activities such as using the toilet) becomes increasingly difficult for affected patients as the disease progresses. As a result, individuals with dementia require extensive care and supervision [12]. Thus, dementia can lead to nursing home admission [5] and premature death [13]. It is also associated with a tremendous economic burden [14]. For example, according to Wimo et al., the annual global societal cost of dementia equaled about US$1313.4 billion for 55.2 million individuals suffering from dementia (US$23,796 per individual with dementia) [14]. Considering the various adverse consequences of dementia, investigating the determinants of dementia is of great importance to policy, clinical practice, and the well-being of individuals with dementia and their families.

As outlined by previous systematic reviews/meta-analyses [15, 16], while there is research that investigates the factors leading to dementia among middle-aged or older adults, there is very limited knowledge regarding the determinants of dementia exclusively among the *oldest old* (e.g., [17–20] *and* additionally based on *longitudinal*, *representative data and* including individuals residing in *institutionalized settings*).

Most of the existing representative studies among the oldest old focused almost exclusively on community-dwelling individuals (and thus not included individuals living in nursing or old age homes) and are often limited by their cross-sectional design. For example, one cross-sectional study [20] based on representative data from the “Mexican Health and Aging Study” and the “Hispanic Established Populations for the Epidemiologic Study of the Elderly” found that higher age and multiple cardiovascular conditions were associated with higher odds of probable dementia among the oldest old living in private households. Other research among the oldest old (e.g., in Calabria, Southern Italy [21]) was also restricted by, among other things, the cross-sectional design (and the almost complete exclusion of nursing home residents). However, longitudinal studies are required to get a better understanding of the determinants of dementia among this important population group. Moreover, it is important to examine the determinants of dementia exclusively in the age bracket of 80 years and above. This can be explained by the fact that some critical life events can occur among individuals aged 80 years and above – such as the death of a spouse or close friend, increasing feelings of loneliness or decline in both mental and physical health.

Overall, due to the limited knowledge in this area, our current study aimed to investigate the determinants of dementia among the *oldest old* using *longitudinal data* from a *representative sample* covering both *community-dwelling and institutionalized individuals*. Such knowledge is important to better understand the factors contributing to dementia over time. This knowledge, in turn, is important to reduce the economic burden associated with dementia and maintain autonomy and satisfaction with life among the oldest old. Ultimately, this may guide efforts to avoid or postpone the onset and progression of dementia in the latest life.

Guided by former research [22, 23] and theoretical considerations, we selected sociodemographic and health-related factors for our regression model (more details are presented in the materials and methods section). For example, education was included as independent variable based on the cognitive reserve hypothesis or brain reserve capacity [24]. This hypothesis refers to the ability to withstand changes in the brain caused by aging and disease without displaying any clinical symptoms or disease indicators [25]. The cognitive reserve can take different forms according to Stern [26]: The neural reserve, where brain networks are more efficient, or have greater capacity, may be less prone to disruption. Furthermore, neuronal compensation, in which alternative networks can balance for the disruption of already existing networks. Moreover, factors such as a smaller network size or loneliness may be associated with a lower likelihood of dementia. Such a link may be explained by social activities and social engagement [27]. According to vascular hypothesis, social engagement can reduce cardiovascular risk factors which attenuates the risk of neurodegenerative diseases [27]. Other authors also attribute the link of social activities and dementia risk to the cognitive reserve hypothesis [28, 29]. Factors such as health literacy may have a long-lasting impact on cognitive impairment in the later stages of life. This may be explained by a more favorable lifestyle [30]. Additionally, depression may also increase the risk of dementia. Such an association may be, among other things, attributed to vascular diseases, changes in alterations in glucocorticoid steroid levels and hippocampal atrophy as well as changes in inflammation [31].

## Materials and methods

### Sample

Longitudinal data (wave 1 and wave 2) from the “Survey on quality of life and subjective well-being of the very old in North Rhine-Westphalia (NRW80+)” were used in this study. The NRW80 + includes individuals over the age of 80 who live in North Rhine-Westphalia, Germany’s most populous state. The distribution of socio-demographic data in North Rhine-Westphalia is very similar compared to the German population as a whole. Various topics are included in the NRW80 + study such as quality of life, socioeconomic issues or health-related factors.

Based on nearly 100 communities in North Rhine Westphalia, a representative sample was drawn. Men and individuals aged 85 years and over were oversampled. Thus, sampling weights were used in regression analysis to account for the complex survey design and to compensate for attrition. The first wave took place between August 2017 and February 2018. In the second wave, data collection among the panel sample (i.e., individuals who already took part in wave 1) took place from June 2019 to February 2020. The average duration for face-to-face interviews was 80 to 90 min. In the first wave, the response rate was 23.4%. However, sociodemographic factors such as age group, living situation and sex were not linked to the likelihood of participation [32]. In the second wave, the response rate was 56.9% in the panel sample. In the provided dataset, individuals from wave 1 were only included when they also completed wave 2. The general dataset and further details can be found here [33].

In our analytic sample, n equaled 1,296 observations (wave 1: 667 individuals; wave 2: 629 individuals). More details are given by Wagner et al. [32].

Informed consent was obtained from all participants or their legal representatives. The NRW80 + was approved by the ethics committee of the Medical Faculty of the University of Cologne (No. 17–169).

### Outcome: dementia

DemTect [34, 35] was used to measure probable dementia. This tool consists of five subtests: Word list/delayed recall, number transcoding, verbal fluency, digit span reverse, and word list delayed recall. A detailed description of the subtests is given by Kalbe and Kessler [36]. The score ranges from 0 to 18, with higher values indicating less cognitive impairment. Dementia is indicated by a score less than 9, whereas values of 9 or higher indicate the absence of dementia. According to Kalbe et al. [34], the DemTect has been shown to be effective in screening for dementia (specificity: 97%; sensitivity: 85.1%).

### Independent variables

The selection of independent variables was guided by former research in this area (for example: [22, 23]) and theoretical considerations. Thus, we included *sociodemographic* and *health-related* factors in regression analysis as follows:

With regard to sociodemographic factors, age (in years), sex (men; women), education (ISCED-97 classification [37]: low, medium, and high education), marital status (married, living together with spouse; Other: single, widowed, divorced, married, living separated from spouse), social network size and loneliness (from 1 = never/almost never to 4 = almost or almost always; higher values thus reflect higher loneliness levels) were included in regression analysis. The loneliness tool is highly correlated with the UCLA loneliness scale [38]. Additionally, we included health-related factors as follows: health literacy (score was built by averaging two items namely knowledge and compliance in the area of health literacy; scores ranges from 1 to 4, higher values reflect higher health literacy), functional impairment, depressive symptoms, and a count score for chronic conditions.

Functional impairment was quantified based on a tool to measure activities of daily living – an amended version of Katz et al. [39]. The tool to quantify functional impairment had seven items, each with three response categories: 0 = only possible with help, 1 = a little help, and 2 = no help needed. The following domains were covered: eating, dressing/undressing, personal hygiene, walking, getting up from bed and lying down, bathing/showering, reaching the toilet in time. The items were averaged and subsequently the coding was reversed. This means that the final score ranges from 0 to 2 whereby higher values reflect higher functional impairment. The “depression in old age scale” (DIA-S) [40, 41] was used to assess depressive symptoms. This tool has four items (each case: no or yes). A sum score was generated using these four items. The sum score ranges from 0 to 4 (higher values reflect more depressive symptoms). Favorable psychometric properties have been shown [40, 41]. To quantify chronic conditions, a count score was generated. To this end, these 19 self-reported chronic illnesses were included (in each case: 0 = absence and 1 = presence): myocardial infarction, heart failure, hypertension, stroke, mental illness, cancer, diabetes, respiratory or pulmonary disease, back pain, gastric or intestinal disease, kidney disease, liver disease, blood disease, joint or bone disease, bladder disease, sleep disorder, eye disease or visual disorder, ear disease or hearing impairment, and neurological disease.

### Statistical analysis

Sample characteristics were first calculated (also stratified by probable dementia). Thereafter, a logistic random-effects (RE) model was estimated (outcome with two categories: individuals without probable dementia, and individuals with probable dementia). Of note, logistic RE models both use between- and within-variation over time. This is a common and widely used panel-econometric model [42]. Regressions were also computed stratified by sex. Statistical analyses were performed using Stata 16.1 (Stata Corp., College Station, Texas).

## Results

### Sample characteristics

In Table 1, sample characteristics (for the analytic sample; pooled over both waves) stratified by probable dementia are given. This means that the data are combined from wave 1 and wave 2. The analytic sample consisted of 1,296 observations (including 754 individuals). Overall, 35 individuals developed dementia from wave 1 to wave 2.

**Table 1:** Sample characteristics for the analytical sample stratified by probable dementia (pooled over both waves, *n* = 1,296 observations)

	Absence of probable dementia	Presence of probable dementia	*p*-value	Total
	*N* = 1210	*N* = 86		*N* = 1296
Age: Mean (SD)	86.2 (4.2)	87.6 (4.0)	< 0.01	86.3 (4.2)
Sex: N (%)			0.04	
Men	649 (94.7%)	36 (5.3%)		685 (100.0%)
Women	561 (91.8%)	50 (8.2%)		611 (100.0%)
Marital status: N (%)			< 0.01	
Married, living separated from spouse; widowed; divorced; single	688 (91.5%)	64 (8.5%)		752 (100.0%)
Married, living together with spouse	522 (96.0%)	22 (4.0%)		544 (100.0%)
Living situation: N (%)			< 0.01	
Private living	1,171 (94.0%)	75 (6.0%)		1,246 (100.0%)
Living in institutionalized settings	39 (78.0%)	11 (22.0%)		50 (100.0%)
Educational level: N (%)			< 0.001	
Low	204 (84.0%)	39 (16.0%)		243 (100.0%)
Medium	646 (94.4%)	38 (5.6%)		684 (100.0%)
High	360 (97.6%)	9 (2.4%)		369 (100.0%)
Size of the social network: Mean (SD)	8.4 (7.9)	5.0 (4.0)	< 0.001	8.2 (7.7)
Loneliness: Mean (SD)	1.3 (0.6)	1.4 (0.6)	0.08	1.3 (0.6)
Health literacy: Mean (SD)	3.7 (0.5)	3.5 (0.7)	< 0.001	3.7 (0.5)
Functional impairment: Mean (SD)	0.2 (0.3)	0.5 (0.5)	< 0.001	0.2 (0.4)
Depressive symptoms: Mean (SD)	0.7 (1.0)	0.8 (1.0)	0.46	0.7 (1.0)
Count score for chronic conditions: Mean (SD)	3.4 (2.2)	3.4 (2.1)	0.87	3.4 (2.2)

*Note* Educational level was quantified using the ISCED-97 classification

Loneliness: Ranging from 1 = never/almost never to 4 = almost or almost always, with higher values indicating higher loneliness

Health literacy: Ranging from 1 to 4, with higher values reflecting higher levels of health literacy

Functional impairment (ADL): Ranging from 0 to 2, with higher values corresponding to higher functional impairment

Depressive symptoms (DIA-S4): Ranging from 0 to 4, with higher values corresponding to more depressive symptoms

Count score for chronic conditions: Ranging from 0 to 19, with higher values corresponding to more chronic conditions

Please note that living situation was not included as independent variable because it may reflect the consequence (rather than the cause) of dementia

In the total sample, average age was 86.3 years (SD: 4.2 years), with 47% being female. Additionally, 18.7% of the individuals had a low education (medium education: 52.8%; high education: 28.5%). Moreover, 6.6% (86 out of 1,296 observations) had probable dementia. Significant differences exist between individuals without probable dementia and individuals with probable dementia in terms of age, sex, marital status, educational level, size of the social network, health literacy, and functional impairment. More details are shown in Table 1.

### Regression analysis

Results of multiple logistic regressions are shown in Table 2 (second column: among the total sample; third column: among men; fourth column; among women). 

**Table 2:** Determinants of probable dementia. Results of logistic RE regressions

	(1)	(2)	(3)
Independent variables	Probable dementia – total sample	Probable dementia – men	Probable dementia - women
Sex: Women (Ref.: Men)	0.68		
	(0.21–2.20)		
Age	1.02	1.10	1.00
	(0.92–1.15)	(0.88–1.37)	(0.88–1.13)
Marital status: Married, living together with spouse (Ref.: Married, living separated from spouse; widowed; divorced; single)	0.44	2.54	0.07*
	(0.14–1.42)	(0.35–18.28)	(0.01–0.54)
Educational level: - low (Ref.: medium)	3.31*	2.94	3.19+
	(1.10–9.98)	(0.13–64.61)	(0.97–10.47)
- high	0.08**	0.11+	0.05+
	(0.01–0.53)	(0.01–1.01)	(0.00–1.31)
Social network size	0.87**	0.93	0.84**
	(0.79–0.96)	(0.81–1.06)	(0.73–0.96)
Loneliness	0.85	0.94	0.97
	(0.41–1.76)	(0.25–3.54)	(0.43–2.21)
Health literacy	0.29***	0.34+	0.24**
	(0.14–0.60)	(0.10–1.14)	(0.10–0.59)
Functional impairment	13.45***	8.72*	18.58***
	(3.86–46.92)	(1.03–73.80)	(3.85–89.69)
Depressive symptoms	0.69	1.24	0.51*
	(0.44–1.09)	(0.55–2.84)	(0.30–0.87)
Count score for chronic conditions	0.86	0.91	0.84
	(0.71–1.05)	(0.62–1.32)	(0.67–1.05)
Observations	1,296	685	611
Number of Individuals	754	396	358

*Notes* Odds Ratios are reported; 95% CI in parentheses; weights were applied; *** *p* < 0.001, ** *p* < 0.01, * *p* < 0.05, + *p* < 0.10

Educational level was quantified using the ISCED-97 classification

Loneliness: Ranging from 1 = never/almost never to 4 = almost or almost always, with higher values indicating higher loneliness

Health literacy: Ranging from 1 to 4, with higher values reflecting higher levels of health literacy

Functional impairment (ADL): Ranging from 0 to 2, with higher values corresponding to higher functional impairment

Depressive symptoms (DIA-S4): Ranging from 0 to 4, with higher values corresponding to more depressive symptoms

Count score for chronic conditions: Ranging from 0 to 19, with higher values corresponding to more chronic conditions

In the total sample, a higher likelihood of probable dementia was positively associated with lower education (e.g., low education compared to medium education: OR: 3.31 [95% CI: 1.10–9.98]), a smaller network size (OR: 0.87 [95% CI: 0.79–0.96]), lower health literacy (OR: 0.29 [95% CI: 0.14–0.60]), higher functional impairment (OR: 13.45 [3.86–46.92]). However, a higher likelihood of probable dementia was not significantly associated with sex, age, marital status, loneliness, and depressive symptoms.

Among men, a higher likelihood of probable dementia was only significantly positively associated with functional impairment (OR: 8.72 [95% CI: 1.03–73.80]). Among women, a higher likelihood of probable dementia was positively associated with not being married (compared to being married, OR: 0.07 [95% CI: 0.01–0.54]), a smaller network size (OR: 0.84 [95% CI: 0.73–0.96]), lower health literacy (OR: 0.24 [95% CI: 0.10–0.59]), higher functional impairment (OR: 18.58 [3.85–89.69]), and fewer depressive symptoms (OR: 0.51 [95% CI: 0.30–0.87]).

In a sensitivity analysis (see Table 3), functional impairment was removed from the model because of the unclear directionality. The results of this model are mostly very similar (in terms of effect size and significance) when compared to the results presented in Table 2. However, the association between low education and likelihood of probable dementia is somewhat stronger (low education compared to medium education: OR: 5.53 [95% CI: 1.74–17.59]), particularly in women (OR: 6.20 [95% CI: 1.83 to 21.03]).

**Table 3:** Determinants of probable dementia. Results of logistic RE regressions (without functional impairment)

	(1)	(2)	(3)
Independent variables	Probable dementia – total sample	Probable dementia – men	Probable dementia - women
Sex: Women (Ref.: Men)	0.70		
	(0.21–2.31)		
Age	1.07	1.14	1.04
	(0.96–1.20)	(0.92–1.42)	(0.92–1.18)
Marital status: Married, living together with spouse (Ref.: Married, living separated from spouse; widowed; divorced; single)	0.43	1.83	0.11*
	(0.13–1.40)	(0.28–11.84)	(0.02–0.72)
Educational level: - low (Ref.: medium)	5.53**	2.29	6.20**
	(1.74–17.59)	(0.11–48.14)	(1.83–21.03)
- high	0.09*	0.13+	0.04+
	(0.01–0.58)	(0.02–1.08)	(0.00–1.09)
Social network size	0.87**	0.93	0.83**
	(0.79–0.96)	(0.82–1.06)	(0.72–0.95)
Loneliness	0.86	0.95	0.93
	(0.42–1.77)	(0.26–3.41)	(0.41–2.09)
Health literacy	0.23***	0.33+	0.19***
	(0.11–0.49)	(0.10–1.07)	(0.08–0.45)
Depressive symptoms	0.77	1.25	0.61+
	(0.49–1.19)	(0.56–2.80)	(0.37–1.01)
Count score for chronic conditions	0.97	1.02	0.94
	(0.80–1.17)	(0.72–1.45)	(0.76–1.16)
Observations	1,296	685	611
Number of Individuals	754	396	358

*Notes* Odds Ratios are reported; 95% CI in parentheses; weights were applied; *** *p* < 0.001, ** *p* < 0.01, * *p* < 0.05, + *p* < 0.10

Educational level was quantified using the ISCED-97 classification

Loneliness: Ranging from 1 = never/almost never to 4 = almost or almost always, with higher values indicating higher loneliness

Health literacy: Ranging from 1 to 4, with higher values reflecting higher levels of health literacy

Depressive symptoms (DIA-S4): Ranging from 0 to 4, with higher values corresponding to more depressive symptoms

Count score for chronic conditions: Ranging from 0 to 19, with higher values corresponding to more chronic conditions

## Discussion

The aim of this study was to examine the determinants of dementia among the oldest old (also stratified by sex) using longitudinal data from a representative sample. Multiple logistic regressions revealed that a higher likelihood of probable dementia was positively associated with lower education, a smaller network size, lower health literacy, and higher functional impairment, whilst likelihood of probable dementia was not statistically significantly associated with sex, age, marital status, loneliness and depressive symptoms in the total sample. Sex stratified regressions were also reported. In sum, our current study extends our current knowledge because this study exclusively used data from the *oldest old* and is based on *longitudinal*, *representative data* (also including individuals residing in *institutionalized settings*).

Our study showed that a low educational level was associated with a higher likelihood of probable dementia – which is well in line with prior research [43]. Possible explanations for such a link mainly refer to the well-known hypothesis of cognitive reserve or brain reserve capacity [24]. Moreover, lifestyle factors such as dietary habits or an active lifestyle could explain the link between educational level and probable dementia [44] given that education may influence human behavior and lifestyle practices which may promote probable dementia during very old age [45].

Our study also identified an association between a smaller network size and a higher likelihood of probable dementia. This aligns with prior research [46], and possible explanations could include social activities and social engagement (e.g., vascular hypothesis or cognitive reserve hypothesis) – as outlined in the introduction section [27]. Other studies have also emphasized the link between a smaller social network size and higher stress or lower self-worth; factors that can contribute to dementia [47].

Interestingly, lower health literacy was also associated with a higher likelihood of probable dementia in our study. This supports the findings of a previous systematic review and may be attributed to a more favorable lifestyle [30]. Lastly, the clear association between functional impairment and probable dementia supports the vast majority of studies (e.g., [48]). Initial problems with ADL may indicate neurodegenerative processes, often with subsequent clinically recognizable dementia [48].

With regard to the potential gender differences identified in our study, it may be worth noting that the results differ in terms of significance. This may be partly explained by differences in the number of cases with dementia between women and men (see Table 1). The signs and effect sizes are often comparable. Different biological (e.g., metabolic) processes may explain some differences, which could be directly related to cognitive function and the etiology of dementia in old age [49, 50]. In this case, different conditions may trigger dementia differently in older men than in their female counterparts [49, 50]. Moreover, particularly the significant link between *more* depressive symptoms and a *lower* likelihood of probable dementia exclusively among women is a bit puzzling – and not in accordance with prior literature [51]. It could be a random effect or it could be related to certain care measures imposed on individuals with depressive symptoms, either in clinical or social settings. For example, those women with more depressive symptoms may be guided through activities to manage the condition, which could indirectly reduce the likelihood of developing dementia. However, this is a speculative explanation and further research is required to examine this association in further detail.

Some advantages and shortcomings in this study should be mentioned: For this study, a large, longitudinal dataset from individuals aged 80 and up was used. Individuals living in both private households and institutionalized settings were included. The response rate was rather low. Thus, certain groups (such as individuals with very poor health or very low education) may have a lower likelihood of participation. However, overall it should be acknowledged that this study is representative of people aged 80 in North Rhine-Westphalia (Germany) [32]. Additionally, sampling weights were used in this study. Established tools were used to quantify the independent variables. Moreover, a screening tool was used to measure cognitive impairment – thus, future research with more sophisticated tools is required to confirm our current findings.

In summary, our study identified factors associated with dementia among the oldest old. Efforts to increase, among other things, formal education, network size, and health literacy may be fruitful to postpone dementia, particularly among women. For example, developing health literacy programs may be beneficial to reduce the burden associated with dementia.

## Data Availability

The NRW 80+ data are available via gesis. For interested researchers, please see: https://search.gesis.org/research_data/ZA7558.
